# Noninvasive Neuroprosthesis Promotes Cardiovascular Recovery After Spinal Cord Injury

**DOI:** 10.1007/s13311-021-01034-5

**Published:** 2021-03-31

**Authors:** Rahul Sachdeva, Tom E. Nightingale, Kiran Pawar, Tamila Kalimullina, Adam Mesa, Arshdeep Marwaha, Alison M. M. Williams, Tania Lam, Andrei V. Krassioukov

**Affiliations:** 1 International Collaboration On Repair Discoveries (ICORD), University of British Columbia, Vancouver, Canada; 2 Department of Medicine, Division of Physical Medicine and Rehabilitation, University of British Columbia, Vancouver, Canada; 3 School of Sport, Exercise and Rehabilitation Sciences, University of Birmingham, Birmingham, UK; 4 School of Kinesiology, University of British Columbia Vancouver, British Columbia Vancouver, British Columbia, Canada; 5 GF Strong Rehabilitation Centre, Vancouver Coastal Health, Vancouver, Canada

**Keywords:** Spinal cord injuries, Cardiovascular dysfunction, Blood pressure, Autonomic dysreflexia, Transcutaneous stimulation, Noninvasive neuromodulation

## Abstract

Spinal cord injury (SCI) leads to severe impairment in cardiovascular control, commonly manifested as a rapid, uncontrolled rise in blood pressure triggered by peripheral stimuli—a condition called autonomic dysreflexia. The objective was to demonstrate the translational potential of noninvasive transcutaneous stimulation (TCS) in mitigating autonomic dysreflexia following SCI, using pre-clinical evidence and a clinical case report. In rats with SCI, we show that TCS not only prevents the instigation of autonomic dysreflexia, but also mitigates its severity when delivered during an already-triggered episode. Furthermore, when TCS was delivered as a multisession therapy for 6 weeks post-SCI, the severity of autonomic dysreflexia was significantly reduced when tested in the absence of concurrent TCS. This treatment effect persisted for at least 1 week after the end of therapy. More importantly, we demonstrate the clinical applicability of TCS in treatment of autonomic dysreflexia in an individual with cervical, motor-complete, chronic SCI. We anticipate that TCS will offer significant therapeutic advantages, such as obviating the need for surgery resulting in reduced risk and medical expenses. Furthermore, this study provides a framework for testing the potential of TCS in improving recovery of other autonomic functions such lower urinary tract, bowel, and sexual dysfunction following SCI.

**Supplementary Information:**

The online version contains supplementary material available at 10.1007/s13311-021-01034-5.

## Introduction

Spinal cord injury (SCI) disrupts crucial communication between spinal and supraspinal autonomic neural circuits, resulting in severely impaired cardiovascular control [1]. Cardiovascular dysfunction affects the majority of individuals with cervical or high-thoracic SCI (≥ T6 spinal segment) and commonly manifests as autonomic dysreflexia, a condition where systolic blood pressure (BP) can abruptly rise often reaching beyond 300 mmHg [2]. Autonomic dysreflexia is commonly instigated by stimuli from activities of daily living (e.g., bowel routine) [3] or iatrogenic medical procedures (e.g., urodynamic evaluations) [4]. These episodes negatively impact the quality of life, interfere with clinical procedures, and can be life-threatening emergencies [5]. Alarmingly, the sporadic episodes of autonomic dysreflexia can occur more than 40 times/day [6] and can lead to myocardial infarction [7] and stroke [8]. Overall, cardiovascular complications are among the leading causes of morbidity and mortality following SCI [9] and regaining cardiovascular control is an urgent unmet need in this population [10].

Despite the detrimental consequences, recovery of cardiovascular function following SCI remains an underexplored research avenue when compared with motor function. Moreover, the majority of pre-clinical interventions have failed to demonstrate clinical efficacy [11]. Furthermore, recovery becomes even more challenging in individuals with a more refractory, chronic SCI [12].

The ability of electrical interfaces to activate disconnected neuronal circuits has gained overwhelming attention in recent years. Epidural stimulation of the injured spinal cord has shown restoration of voluntary motor control in completely paralyzed humans [13] as well as in rodent models [14]. Similarly, improvements in cardiovascular function following SCI have also been reported with epidural stimulation [15–17]. While promising, a major drawback of epidural stimulation is the need for an invasive and expensive surgical procedure. Furthermore, the inability to re-position an implanted electrode considerably limits the adaptability of this procedure across various spinal levels to target specific functions.

We have demonstrated that non-invasive transcutaneous stimulation (TCS) of the spinal cord can ameliorate orthostatic hypotension in individuals with SCI [18]. However, whether TCS can mitigate autonomic dysreflexia episodes remains to be tested. Here, we used a translational approach to examine the efficacy of TCS in a rat model of chronic SCI and to validate its clinical feasibility in an individual with SCI. We hypothesized that acute TCS will prevent the instigation of autonomic dysreflexia despite the noxious trigger being present and will also interrupt autonomic dysreflexia when initiated during an ongoing hypertensive episode. Furthermore, we tested whether long-term TCS mitigates autonomic dysreflexia, when tested independently of active TCS. Finally, we examined the clinical adaptability of TCS as an acute therapeutic intervention for autonomic dysreflexia in an individual with chronic, motor-complete cervical SCI.

## Methods

### Pre-clinical Rat Model

**Fig. 1:**
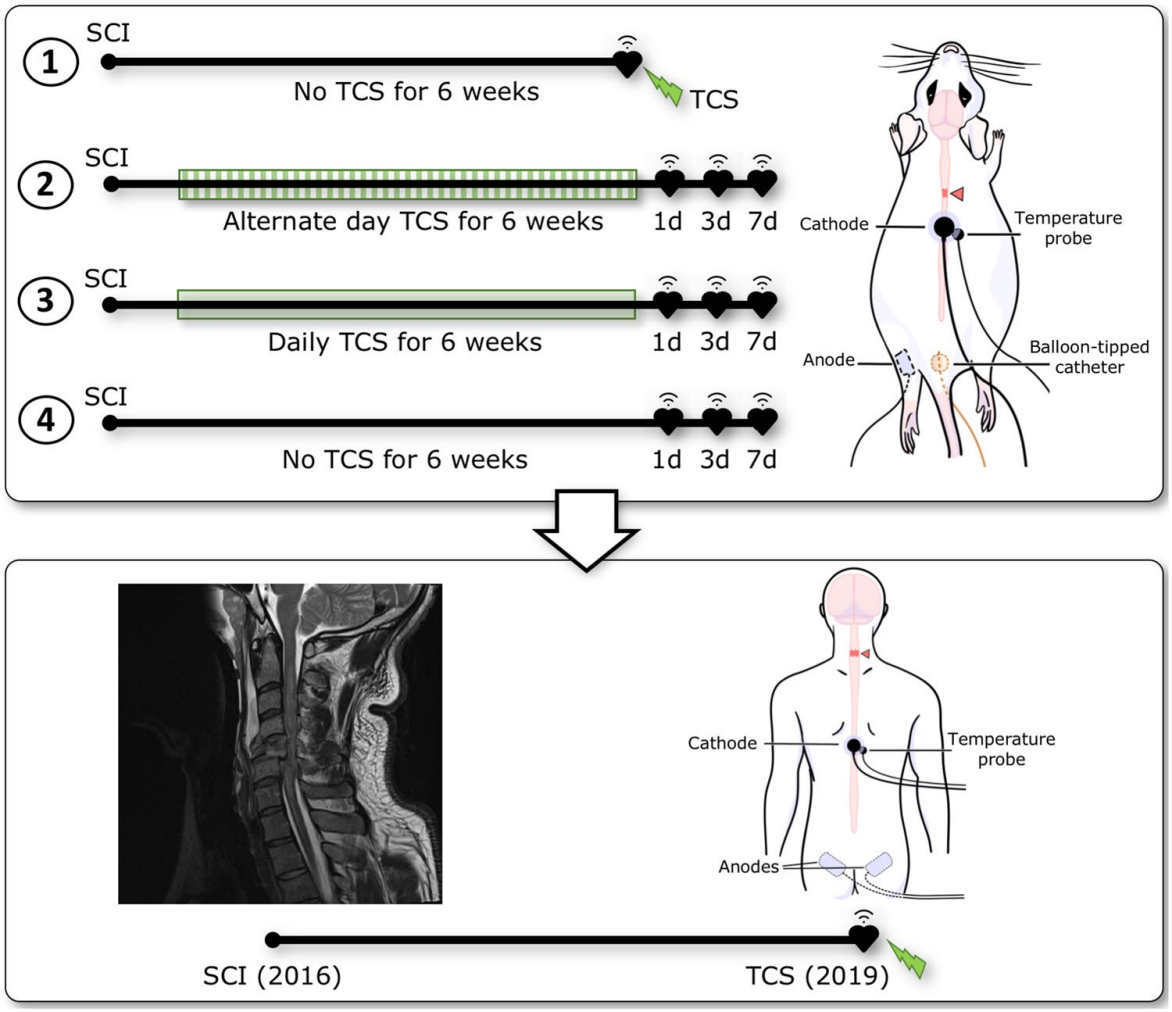
Overview of the translational approach. *Upper panel* represents individual timelines for animal experiments showing four animal groups. A schematic drawing of a rat shows the experimental setup of cathode and anode for TCS, a temperature probe, and a balloon-tipped catheter for CRD. Black hearts represent wireless cardiovascular assessments. *Lower panel* represents the clinical case. A T2 magnetic resonance imaging in the sagittal plane demonstrates the SCI at C4 level. A timeline shows the TCS experiments delivered at 3 years post-SCI. A schematic drawing shows the experimental setup for a stimulating cathode with a skin temperature probe and two anodes. Please refer to main text for additional details

The procedures were performed in compliance with the Canadian Council on Animal Care guidelines, with protocols approved by the Animal Care Committee at the University of British Columbia (approval certificate: A18-0183). Forty-three adult (~ 300 g), male Wistar rats (Harlan Laboratories, USA) received a complete transection at the T3 spinal segment and were divided across four groups (Fig. 1). Group 1 (*n* = 13) was utilized for acute TCS experiments and groups 2, 3, and 4 (*n* = 10/group) were used in the long-term TCS experiments.

#### SCI Procedure

A laminectomy was performed to expose the T3 spinal segment. Dura mater was incised and the spinal cord was completely transected using micro-scissors [19]. Dura mater was closed using 9-0 sutures, followed by closure of muscle and skin using 4-0 sutures [20]. Pre- and post-operative care was performed as described previously [21].

#### Telemeter Implantation

Six weeks post-SCI, rats were implanted with a wireless telemeter (TRM54P; Kaha Sciences, New Zealand). With the rat placed in a supine position, an inguinal skin incision was made to expose the femoral artery. The pressure-sensing tip of the telemeter was inserted and secured in the femoral artery, followed by skin closure using 4-0 sutures.

#### Experimental Autonomic Dysreflexia

Autonomic dysreflexia was induced via colorectal distension (CRD) using a balloon-tipped catheter (AA6110-Coloplast, Canada). Beat-by-beat BP and heart rate (HR) were recorded using an analog-to-digital converter (PowerLab/16SP ML 795, LabChart 8, ADInstruments, USA). The balloon was inflated with 2 mL of air over 10 s, remaining inflated for 60 s. The difference of systolic and diastolic BP (SBP and DBP) and HR between baseline and the peak value during distension was calculated and averaged across two trials per rat.

#### Acute (Short-Term) TCS

Group 1 rats received no TCS until 6 weeks after SCI when the acute, real-time effect of TCS was tested. Stimulations (1 ms, 30 Hz, biphasic pulses, 5–10 mA) were delivered using a Model 2100 Isolated Pulse Stimulator (A-M systems) via cathode (1 cm in diameter; DBF3D77, Biofeedback Resources International, USA) placed at the mid-dorsal surface at the T7 level. Anode (Kendall™ ER88007, McKesson, Canada) was placed ventrally along the femur (Fig. 1). The efficacy of acute TCS was tested across two distinct therapeutic principles. First, the ability of TCS to prevent autonomic dysreflexia was examined by initiating TCS 2–3 min prior to CRD. Second, the ability of TCS to interrupt autonomic dysreflexia was tested by initiating TCS 15 s after achieving full distension.

#### Chronic (Long-Term) TCS

TCS was delivered as a multi-session therapy starting at 5 days post-SCI and continuing for 6 weeks. Stimulations were delivered for 30 min (10 mA, 30 Hz) as two 15-min periods separated by 5 min. Rats were randomly allocated to either alternate day TCS (3 days/week; group 2) or daily TCS (5 days/week; group 3) or no TCS (group 4) groups. One rat died post-surgery, resulting in *n* = 9 for group 4. In order to test the potentially permanent effects of TCS, cardiovascular assessments during CRD were performed at 1, 3, and 7 days after the last stimulation session, independent of active, concurrent TCS. All assessments were performed with personnel blinded to the animal group allocation. Arrhythmia events were manually identified on arterial pressure waveforms. The proportion of rats with arrhythmia and the event frequency (events/CRD episode) were quantified. Local skin temperature and core body temperature were recorded via a skin probe (MLT422, AD Instruments) and a rectal thermometer (20250-91, Cole-Parmer, Canada), respectively.

#### Statistical Analyses

Baseline hemodynamics at rest and autonomic dysreflexia severity within acute TCS group were compared via two-tailed, paired *t* tests. Baseline hemodynamics and autonomic dysreflexia severity between chronic TCS groups was compared via one-way ANOVA (repeated measures were used for comparison within a group at different timepoints), followed by Tukey’s test for multiple comparisons. Proportion of rats with arrhythmia events was compared using the Fisher’s exact test, followed by Pearson chi-square test for pairwise comparisons. Frequency of arrhythmia events were compared across groups using the Kruskal-Wallis test, followed by Dunn’s test for multiple comparisons. Skin and rectal temperatures were compared using one-way repeated measures ANOVA. Data are reported as mean ± standard deviation (SD) with statistical significance at *p* < 0.05.

## Clinical Translation (Case Report)

### Study Design

A 37-year-old man with a chronic (3 years) motor-complete SCI (American Spinal Injury Association Impairment Scale A) at the fourth cervical (C4) level was assessed across three laboratory visits. The participant provided written informed consent. This case study was approved by the University of British Columbia Clinical Ethics Board. The ability of TCS to prevent (5 trials) and interrupt (4 trials) autonomic dysreflexia was tested across visits.

### Outcome Measures

Beat-by-beat BP was measured via finger photoplethysmography (Finapres® NOVA, Finapres Medical Systems, The Netherlands) corrected to brachial BP (Dinamap Pro 300V2, General Electric, USA) throughout examinations. HR was recorded via 3-lead electrocardiography (ML132, ADInstruments). Skin temperature beneath the cathode was monitored using a temperature probe (MLT422, AD Instruments). HR, BP, and skin temperature were sampled at 1000 Hz using an analog-to-digital converter.

### Clinical Autonomic Dysreflexia

Digital anorectal stimulation (DARS) is a routine procedure to initiate a bowel routine and has previously been employed to trigger controlled elevation in BP [22]. DARS was performed by an experienced clinician (AVK) in accordance with recommendations by Coggrave and Norton [23], while both clinician and the participant remaining blinded to the concurrent change in BP. The participant completed a bowel routine within 24 h prior to assessment. The participant laid on his right side and DARS was delivered via an index finger inserted into the rectum, applying gentle pressure for 30–60 s. Considering participant’s safety, the BP was elevated in a controlled manner with DARS being terminated upon participant’s self-perception of autonomic dysreflexia (i.e., feeling flushed, sweating, goose bumps). Surface electromyography (EMG) electrodes (Trigno, Delsys Inc, USA) were placed perianally to bilaterally record from the pelvic floor muscles (PFM) and monitor the influence of DARS on the perineum. EMG data were recorded at 2000 Hz and analyzed offline using custom MATLAB routines (Mathworks, USA). Data were band-stop filtered at 30, 60, 90, and 120 Hz and low-pass filtered at 100 Hz using an 8th order Butterworth filter.

### Acute TCS

TCS was delivered as continuous pulses (2 ms, 30 Hz, 20–30 mA) using an isolated bipolar constant current stimulator (Digitimer DS5, Digitimer Ltd., UK). Current was applied at the T7/8 spinous processes using a circular cathode with a diameter of 30 mm (Canadian Medical Products Ltd., Canada) placed on the skin (Fig. 1). Two 5 × 9 cm anodes (Canadian Medical Products Ltd., Canada) were placed on the skin over iliac crests. To determine the ability of TCS to prevent an episode of autonomic dysreflexia, TCS was applied prior to DARS. Alternatively, to determine the ability of TCS to interrupt an episode of autonomic dysreflexia, TCS was initiated 30 s into the DARS, which remained ongoing for an additional 30 s.

### Statistical Analyses

The magnitude of the change in hemodynamic responses to DARS was calculated as the difference between the average 1 min baseline and the peak value obtained during DARS. Values are displayed as median Δ change and range. Percentage change was calculated to contextualize the magnitude of the differences between TCS OFF and TCS ON.

## Results

### Pre-clinical Study

#### Effect of Acute TCS on Resting Hemodynamics

In order to test whether acute TCS affects resting hemodynamics, beat-by-beat BP and HR were measured 2 min prior to and 2 min after cessation of TCS. HR was elevated by 12 bpm for 2 min after cessation of TCS (*p* = 0.02). There was no lasting effect of acute TCS on BP (Table 1).

#### Acute Prevention of Autonomic Dysreflexia

**Fig. 2:**
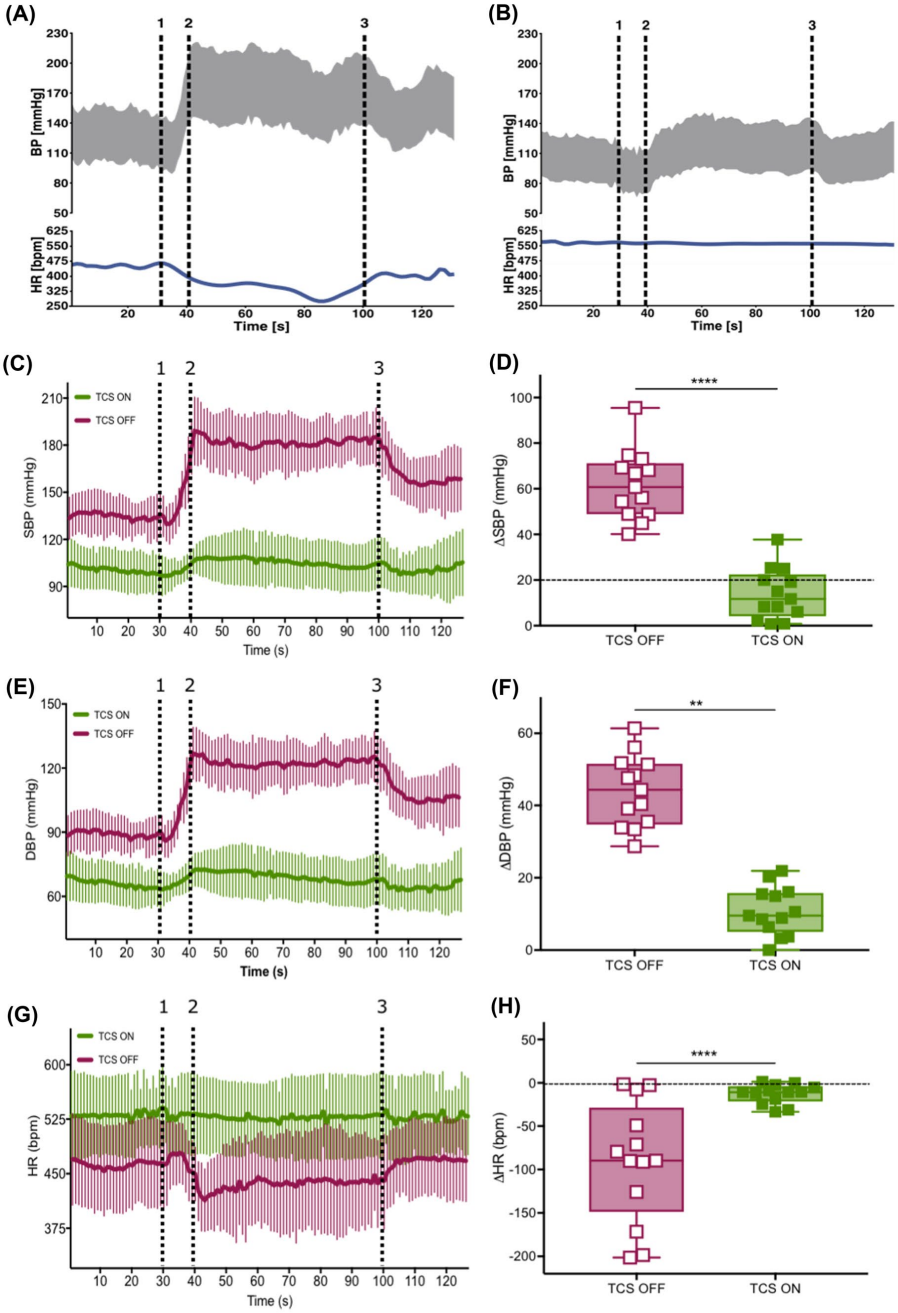
Acute TCS prevents autonomic dysreflexia in rats. **a** Representative manifestation of an episode of autonomic dysreflexia in one rat with chronic SCI (BP-gray, HR-blue) using CRD via a balloon-tipped catheter (1-balloon inflation begins, 2-balloon fully inflates, and 3-balloon is deflated). **b** Response to CRD in the same rat while continuous TCS is turned ON prior to balloon inflation shows prevention of autonomic dysreflexia. **c**, **e**, and **g** Time-locked averages of SBP, DBP, and HR, respectively, during CRD in thirteen rats. **d**, **f**, and **h** Quantification of CRD-dependent maximal change in SBP, DBP, and HR from baseline when TCS is OFF vs ON. Dashed line at 20 mmHg in ΔSBP graph (**d**) represents the clinical definition of autonomic dysreflexia, i.e., rise in SBP by 20 mmHg in response to a peripheral stimulus

In group 1, CRD induced severe autonomic dysreflexia (ΔSBP 62 ± 15 mmHg; Fig. 2a, c, d). However, when TCS was initiated prior to CRD, the same visceral stimulus resulted in a significantly smaller rise in SBP (14 ± 11 mmHg; *p* < 0.0001; Fig. 2b–d). Similarly, there was a preventative effect of TCS on the increase in DBP (44 ± 10 vs. 11 ± 7 mmHg; *p* < 0.0001; Fig. 2e, f) and the concomitant decrease in HR (− 91 ± 69 vs. − 13 ± 11 bpm; *p* = 0.0026; Fig. 2g, h).

Interestingly, when TCS was off, the insertion of a deflated balloon-tipped catheter into the rectum also caused elevation in resting BP (Fig. 2c, e) and concomitant reduction in HR (Fig. 2g). This cardiovascular response to catheter insertion was also prevented by TCS (TCS off vs. on: SBP 134 ± 12 vs. 101 ± 12 mmHg; *p* < 0.0001, DBP 88 ± 8 vs. 67 ± 9 mmHg; *p* < 0.0001, HR 465 ± 55 vs. 529 ± 55 bpm; *p* = 0.0018).

#### Acute Interruption of Autonomic Dysreflexia

**Fig. 3:**
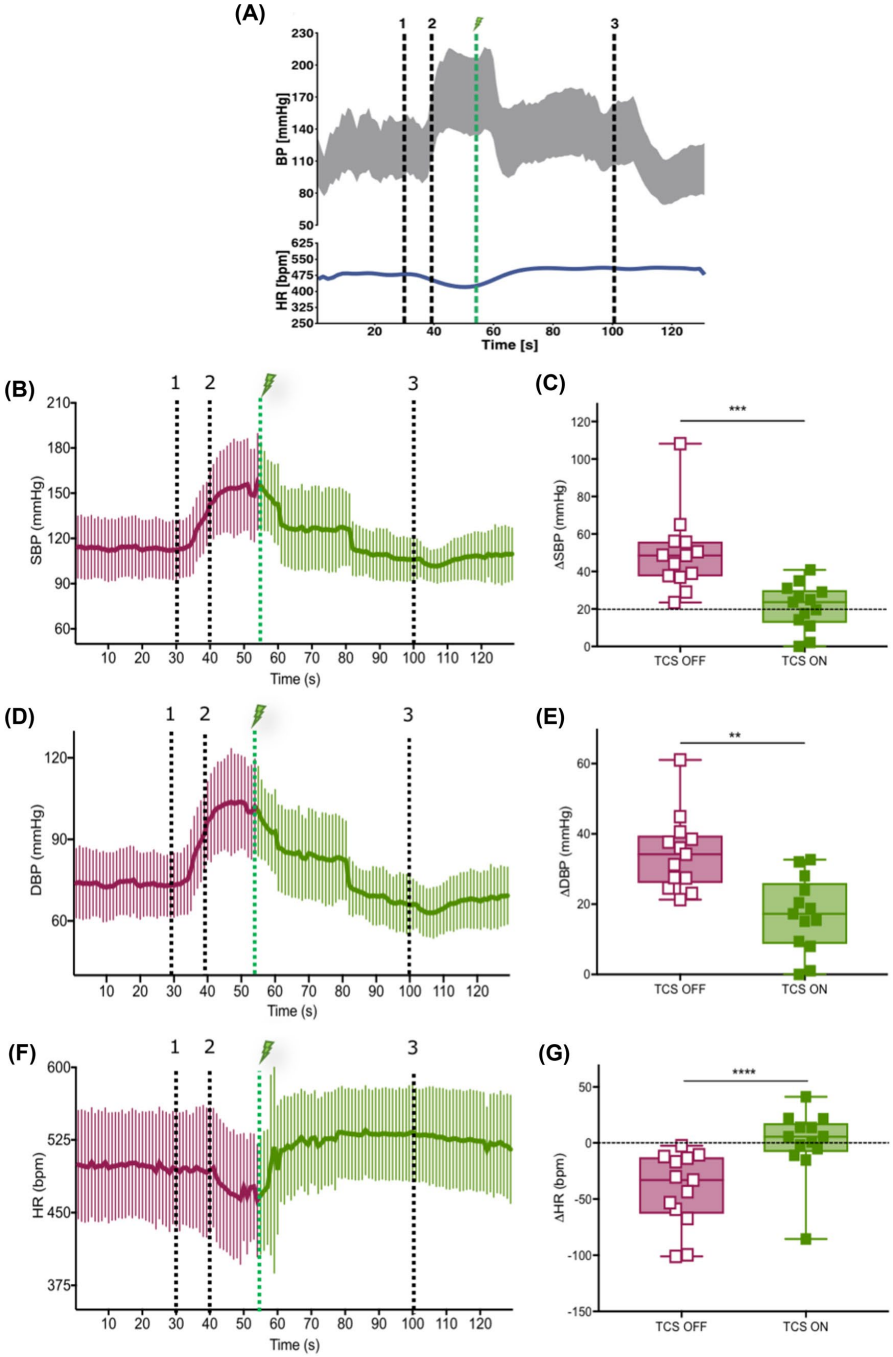
Acute TCS interrupts autonomic dysreflexia in rats. **a** Representative manifestation of an episode of autonomic dysreflexia in one rat with chronic SCI (BP-gray, HR-blue) using CRD being interrupted by TCS (1-balloon inflation begins, 2-balloon fully inflates, lightning bolt-TCS initiated, and 3-balloon is deflated). **b**, **d**, and **f** Time-locked averages of SBP, DBP, and HR, respectively, during similar experiment in thirteen rats. **c**, **e**, and **g** Quantification of CRD-dependent maximal change in SBP, DBP, and HR from baseline when TCS is OFF vs ON. Dashed line at 20 mmHg in ΔSBP graph (**c**) represents the clinical definition of autonomic dysreflexia, i.e., rise in SBP by 20 mmHg in response to a peripheral stimulus

The efficacy of acute TCS to interrupt autonomic dysreflexia was tested by initiating TCS during ongoing CRD in group 1 (Fig. 3a). CRD triggered a rapid increase in SBP (49 ± 21 mmHg) and DBP (34 ± 11 mmHg) with a concomitant decrease in HR (− 42 ± 33 bpm). However, the initiation of TCS immediately reduced the severity of these changes (i.e., ΔSBP 21 ± 12 mmHg, *p* = 0.0002; Fig. 3b, c, ΔDBP 17 ± 11 mmHg, *p* < 0.0001; Fig. 3d, e and ΔHR 0.4 ± 30 bpm, *p* = 0.0029; Fig. 3f, g), despite CRD being maintained.

#### Effect of Chronic TCS on Resting Hemodynamics

Beat-by-beat BP and HR were measured at three timepoints after the cessation of 6-week alt. day or daily TCS. The resting hemodynamics remained largely unaffected by long-term spinal cord stimulations (Table 2). Although one-way repeated measures ANOVA revealed statistically significant effect in DBP, MAP, and HR within alt. day TCS group and HR in daily TCS group, post hoc pairwise comparisons between timepoints showed no statistically significant effect. Similarly, one-way ANOVA showed a significant difference in resting SBP and MAP between the groups on day 3 after the cessation of TCS, but pairwise post hoc comparisons revealed no significant differences. This suggests that the resting hemodynamics remain largely unaltered in response to long-term TCS.

**Table 1:** Baseline cardiovascular measures prior to and after acute TCS. Data are represented as mean ± SD

	Before TCS	After TCS	*P* value
SBP (mmHg)	114.27 ± 20.98	115.30 ± 17.00	0.74
DBP (mmHg)	73.62 ± 13.15	73.75 ± 10.57	0.06
MAP (mmHg)	87.17 ± 15.55	87.60 ± 12.46	0.87
HR (bpm)	497.32 ± 52.90	509.15 ± 59.63	0.02

**Table 2:** Baseline cardiovascular measures after 6 weeks of chronic TCS. Data are represented as mean ± SD

		Day 1	Day 3	Day 7	*P* value (between timepoints)
No TCS	SBP (mmHg)	133 ± 9	130 ± 5	134 ± 5	0.31
	DBP (mmHg)	95 ± 7	91 ± 5	94 ± 7	0.30
	MAP (mmHg)	108 ± 8	104 ± 5	108 ± 6	0.31
	HR (bpm)	527 ± 35	533 ± 47	514 ± 58	0.52
Alt. Day TCS	SBP (mmHg)	138 ± 5	140 ± 9	134 ± 6	0.05
	DBP (mmHg)	98 ± 4	96 ± 7	92 ± 5	0.01
	MAP (mmHg)	111 ± 4	111 ± 8	106 ± 5	0.02
	HR (bpm)	480 ± 45	506 ± 42	520 ± 29	0.03
Daily TCS	SBP (mmHg)	135 ± 8	140 ± 5	144 ± 14	0.09
	DBP (mmHg)	95 ± 5	96 ± 5	98 ± 10	0.44
	MAP (mmHg)	109 ± 6	110 ± 4	114 ± 11	0.24
	HR (bpm)	489 ± 38	543 ± 36	509 ± 48	0.006
*P* value (between groups)	SBP	0.24	0.003	0.037	
	DBP	0.53	0.12	0.17	
	MAP	0.40	0.03	0.09	
	HR	0.03	0.14	0.86	

#### Chronic Treatment of Autonomic Dysreflexia

**Fig. 4:**
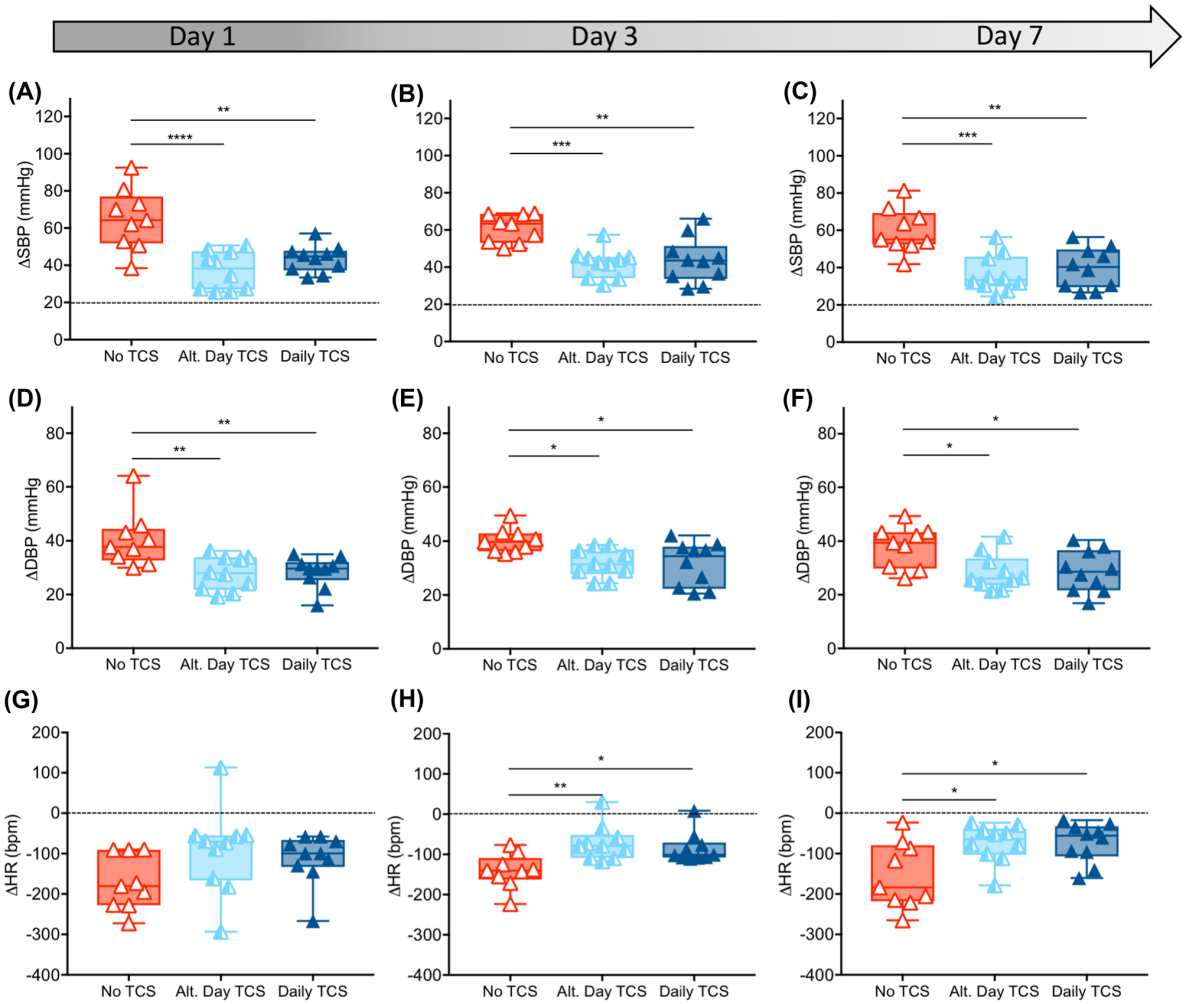
Effects of chronic multisession TCS on autonomic dysreflexia in rats. **a**–**c** Comparison of severity of rise in SBP in response to CRD in groups receiving no TCS, alternate day TCS, and daily TCS at 1, 3, and 7 days post cessation of multi-session TCS therapy. The results show significant effects of TCS on ΔSBP severity regardless of the stimulation paradigm, lasting for 7 days after the end of stimulation. Similar effects were seen in ΔDBP for all three timepoints (**d**–**f**) and ΔHR for days 3 and 7 (**g**–**i**)

Cardiovascular assessments were performed on 1, 3, and 7 days following cessation of TCS (Fig. 4a–i). Group comparisons revealed significant differences in CRD-induced rise in SBP (day 1, *p* < 0.0001; day 3, *p* = 0.0003; and day 7, *p* = 0.0002; Fig. 4a–c) and DBP (day 1, *p* = 0.0019; day 3, *p* = 0.008; and day 7, *p* = 0.014; Fig. 4d–f). Similar comparisons revealed a significant difference in decease in HR at days 3 and 7 but not on day 1 (day 1, *p* = 0.10; day 3, *p* = 0.003; and day 7, *p* = 0.009; Fig. 4g–i). In cases where the group analysis revealed a significant difference, pairwise comparisons revealed that both treatment groups were significantly improved compared with no TCS control (*p* ≤ 0.017) and that there were no differences between the two treatment groups (*p* ≥ 0.46).

#### Mitigation of Arrhythmia

**Fig. 5:**
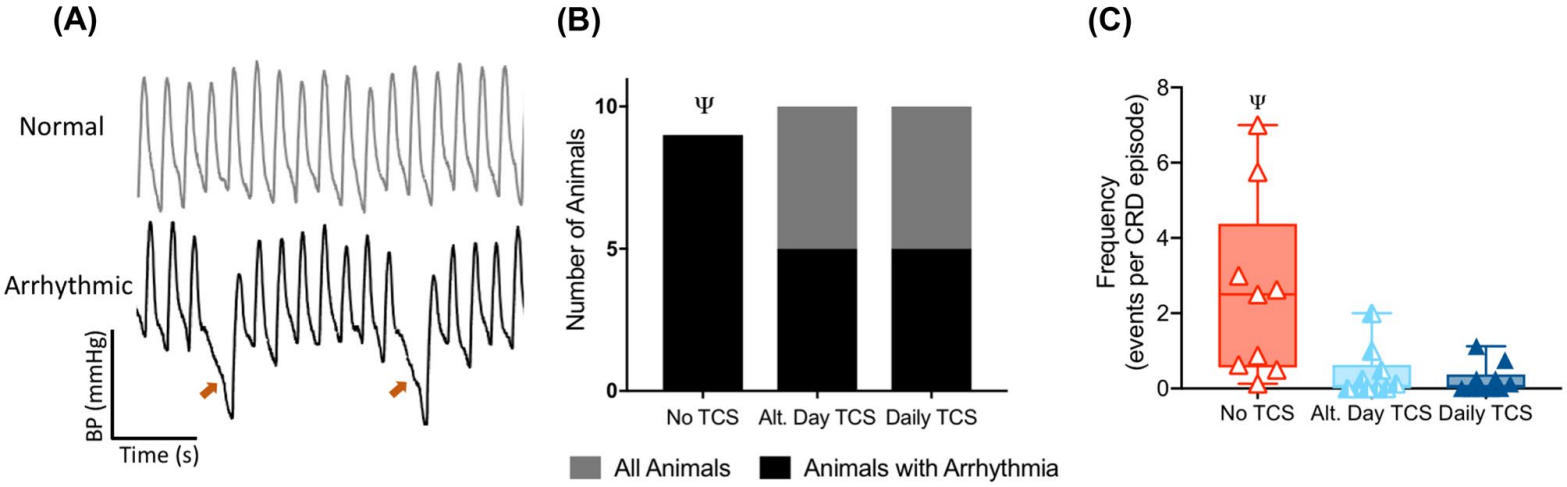
Effects of chronic multisession TCS on cardiac arrhythmia in rats. **a** Representation of a normal arterial pressure waveform (top) and a waveform showing two arrhythmia events (red arrows, bottom). **b** Both TCS paradigms result in a significantly smaller proportion of rats experiencing arrhythmia (Ψ represents significant difference from both treatment groups). **c** Both TCS paradigms resulted in a significantly lower frequency of arrhythmia events during autonomic dysreflexia (Ψ represents significant difference from both treatment groups)

Nine out of nine animals in the control group showed cardiac arrhythmia events during autonomic dysreflexia compared with 5/10 animals in each of the treatment groups (*p* = 0.03; Fig. 5a, b). Pairwise comparisons revealed a greater proportion of rats experiencing arrhythmias in the control group compared with each of the treatment groups (*p* = 0.013). The frequency of arrhythmia events was also different across groups (*p* = 0.003; Fig. 5c), with both treatment groups showing significantly lower arrhythmia frequency compared with control (*p* = 0.01). No difference was seen between the two treatment groups (*p* = 0.90).

#### Temperature Changes During TCS

**Fig. 6:**
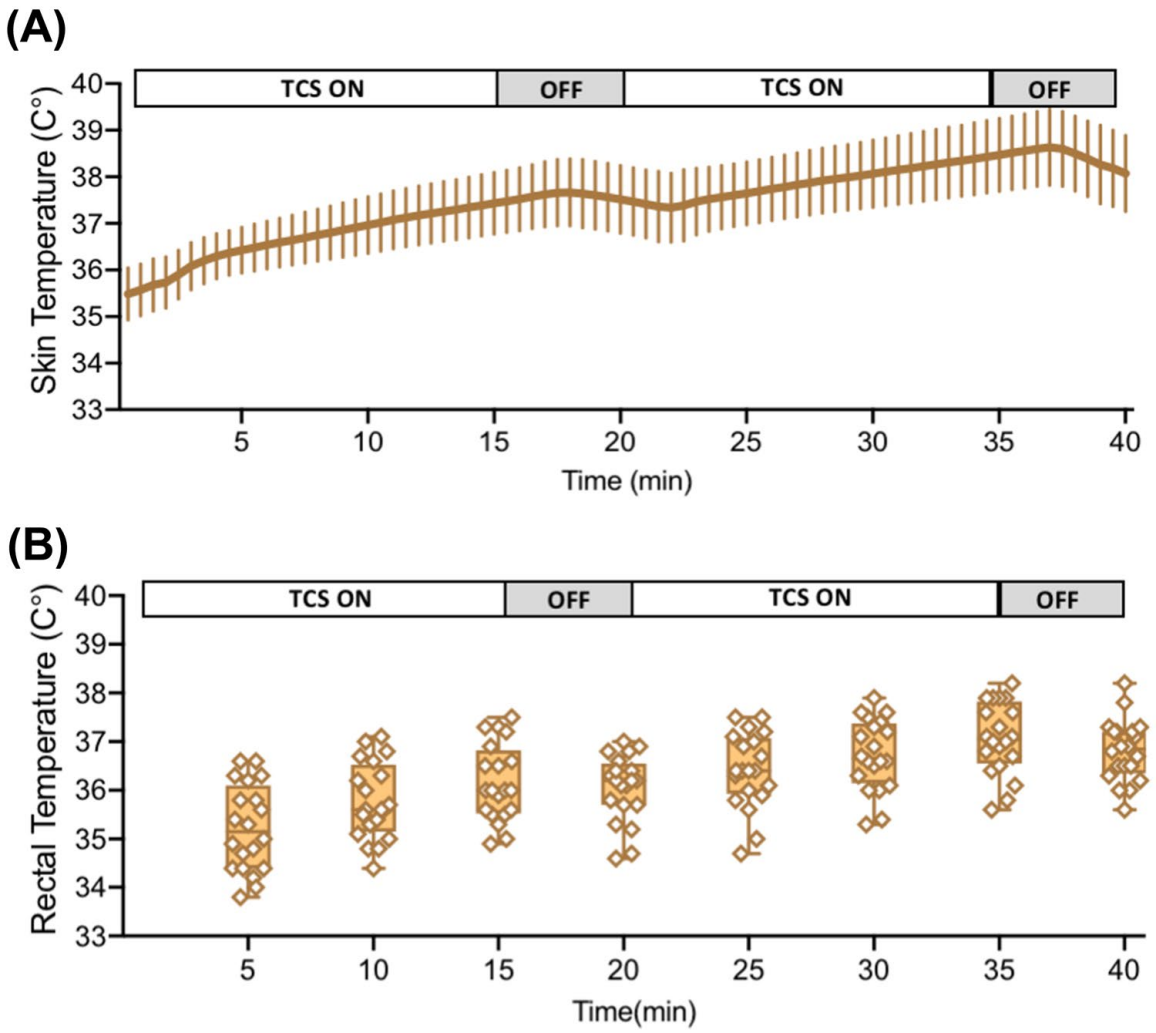
Effects of TCS on skin and core temperature in rats. **a** Continuous recording of skin temperature near the stimulation site during 30 min of TCS. (N) Intermittent readings of rectal temperature during 30 min session of TCS

During the 30 min of TCS, the maximum rise in temperature at the site of stimulation was 3.1 °C (35.5 ± 0.5 to 38.6 ± 0.8 °C; *p* < 0.0001, Fig. 6a) and 1.8 °C (35.2 ± 0.8 to 37.1 ± 0.7 °C; *p* < 0.0001, Fig. 6b) in the core body temperature.

## Clinical Case

### Acute Prevention of Autonomic Dysreflexia

**Fig. 7:**
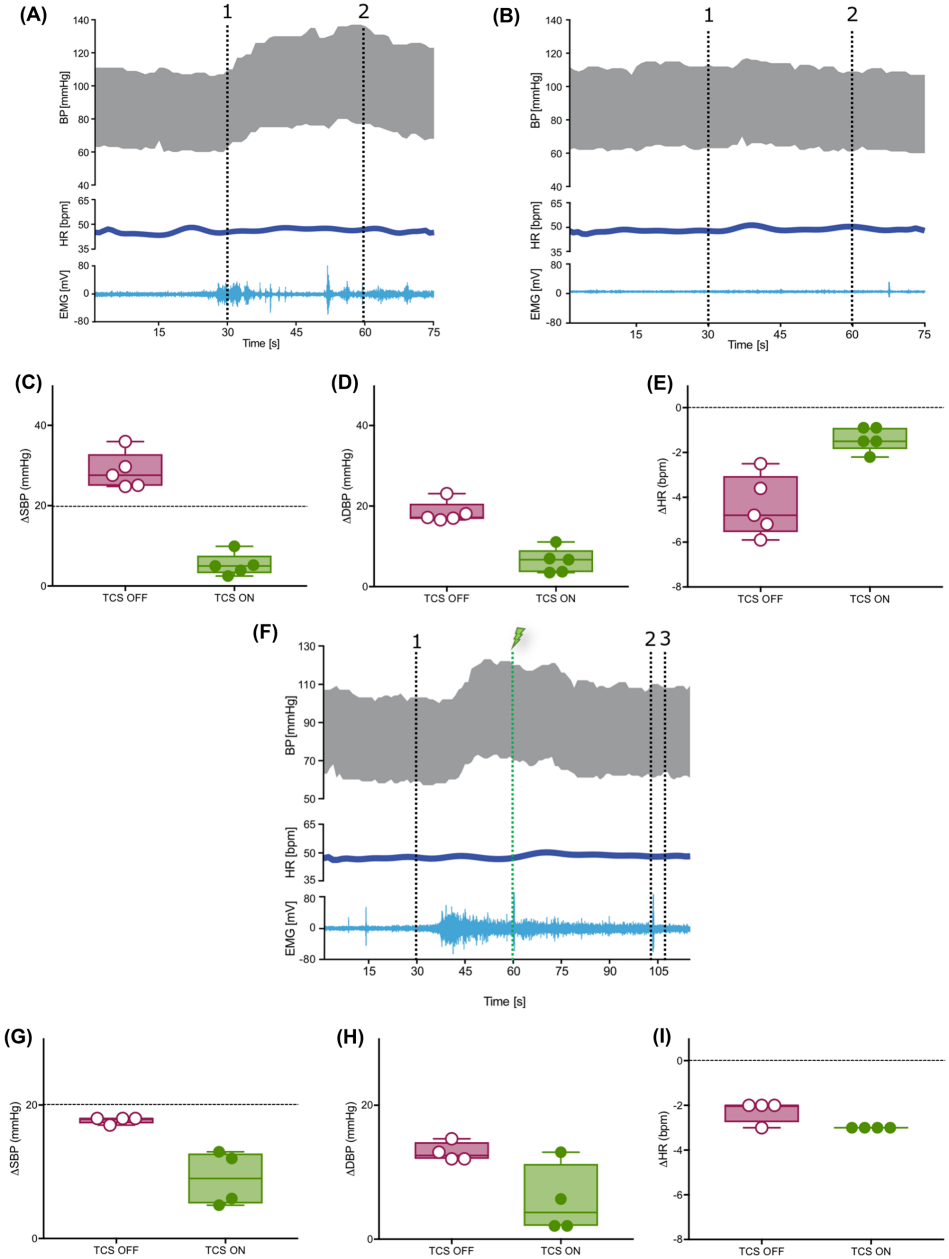
Acute TCS can prevent and interrupt the episodes of autonomic dysreflexia in an individual with SCI. **a** Manifestation of an episode of autonomic dysreflexia in the participant with chronic, motor-complete SCI in response to DARS and in the absence of TCS (1-DARS ON and 2-DARS OFF). **b** Response to DARS in the same participant in the presence of continuous TCS shows prevention of autonomic dysreflexia. **c**, **d**, and **e** Quantification of DARS-dependent maximal change in SBP, DBP, and HR from baseline when TCS is OFF vs ON. Dashed line at 20 mmHg in ΔSBP graph (**c**) represents the clinical definition of autonomic dysreflexia, i.e., rise in SBP by 20 mmHg in response to a peripheral stimulus. **f** DARS triggers severe rise in BP in the absence of TCS. However, elevated BP is normalized when TCS (lightning bolt) is turned on (1-DARS ON and 2-DARS OFF, 3-TCS OFF). **g**, **h**, and **i** Quantification of DARS-dependent maximal change in SBP, DBP, and HR from baseline when TCS is OFF vs ON. EMG traces showing the PFM response to DARS and TCS are shown for each condition (**a**, **c**, and **f**)

DARS resulted in a consistent, controlled rise in BP (ΔSBP 29 ± 5 and ΔDBP 18 ± 3 mmHg) and a reduction in HR (−4 ± 1 bpm), characteristic of autonomic dysreflexia (representative trace presented in Fig. 7a). The small change in HR is likely a result of the controlled rise in BP during DARS as the participant’s comfort and safety was the primary concern. However, when TCS was turned on prior to DARS (representative trace shown in Fig. 7b), the same visceral stimulus resulted in an 82% reduced rise in SBP (5 ± 3 mmHg; Fig. 7c), 65% reduced rise in DBP (6 ± 3 mmHg; Fig. 7d) and 68% lesser reduction in HR (−1 ± 0.5 bpm; Fig. 7e).

### Acute Interruption of Autonomic Dysreflexia

The efficacy of acute TCS to interrupt an episode of autonomic dysreflexia was tested by initiating TCS during an episode of DARS (representative trace shown in Fig. 7f). DARS triggered autonomic dysreflexia in a controlled manner with a rise in BP (ΔSBP 18 ± 1 and ΔDBP 13 ± 1 mmHg) and reduction in HR (−2 ± 0.5 bpm). Initiation of TCS during DARS mitigated autonomic dysreflexia by causing a 49% reduction in SBP (9 ± 4 mmHg; Fig. 7g) and 56% reduction in DBP (6 ± 5 mmHg; Fig. 7h). No TCS-dependent change was seen in HR (−3 ± 0 bpm; Fig. 7i).

### EMG Findings

The application of DARS resulted in expected reflexive PFM activity. However, the presence of TCS appeared to reduce the level of tonic PFM activity.

### Temperature Changes During TCS

No long-term stimulations were delivered to the participant. The duration of continuous TCS ranged between 1 and 4 min. Simultaneous recording of temperature under cathode revealed a maximum rise of 1.3 °C (32.9 to 34.3 °C; data not shown).

## Discussion

The present study shows that acute TCS is an effective strategy for mitigating debilitating episodes of autonomic dysreflexia, with potentially permanent effects achieved via long-term stimulation. Interestingly, alternate day TCS demonstrates similar efficacy as daily TCS, which could be clinically meaningful in reducing the required number of participants’ visits. Long-term TCS also ameliorated cardiac arrhythmias, demonstrating a secondary benefit of substantial clinical importance [24]. Finally, we demonstrate the clinical applicability of TCS by demonstrating TCS-dependent prevention/interruption of autonomic dysreflexia in an individual with chronic, cervical, motor-complete SCI. Previous clinical study by our laboratory utilized monophasic electrical pulses to transcutaneously stimulate the spinal cord and increase BP and alleviate orthostatic hypotension (i.e., low BP) in individuals with SCI. While the spinal cord stimulation paradigm utilized in this prior study is believed to excite propriospinal interneurons and sympathetic preganglionic neurons via sensory afferents [18], biphasic square pulses used in the present study appear to inhibit the sympathetic reflex response resulting in autonomic dysreflexia. This is consistent with the work from Richardson and colleagues that used percutaneous epidural spinal cord stimulation to generate low frequency, low voltage, square wave output to produce an ‘electronic central sympathetic inhibitory mechanism’ to reduce spasticity [25] and autonomic dysreflexia in individuals with SCI [26].

Various pre-clinical approaches have been tested to mitigate autonomic dysreflexia. These broadly include (a) reducing aberrant sprouting of nociceptive sensory afferents [27, 28], (b) minimizing secondary damage [29], and (c) restoring supraspinal control [30, 31]. Most of these strategies have been able to reduce the severity of autonomic dysreflexia by 30–50%. To put this in perspective, a much simpler, noninvasive TCS approach was able to reduce the severity of autonomic dysreflexia by 78% in rats and 82% in a human with chronic SCI. Clinically, pharmacological modulation is among the primary options to manage autonomic dysreflexia [32]. However, a major disadvantage is that most drugs require several minutes to become active and exert prolonged cardiovascular effects [33]. Given the rapid appearance of reflex-driven BP changes in autonomic dysreflexia, spinal cord stimulation may offer a more reliable, immediately acting solution.

Spinal cord stimulation, via invasive means, has shown remarkable potential in improving cardiovascular function in individuals with SCI. Most studies have been focused on mitigating hypotension (i.e., low BP at rest and orthostatic intolerance) following SCI [15–17], with one prior report showing mitigation of autonomic dysreflexia [26]. However, despite the highest surgical standards, surgical procedures have an inherent risk of complications [34], with a risk of further spinal cord damage [35]. TCS may offer similar neuromodulatory effects, without the need for surgical implants. From the safety standpoint, we tested a remote possibility of skin overheating [36], and TCS appeared safe for the durations tested.

**Fig. 8:**
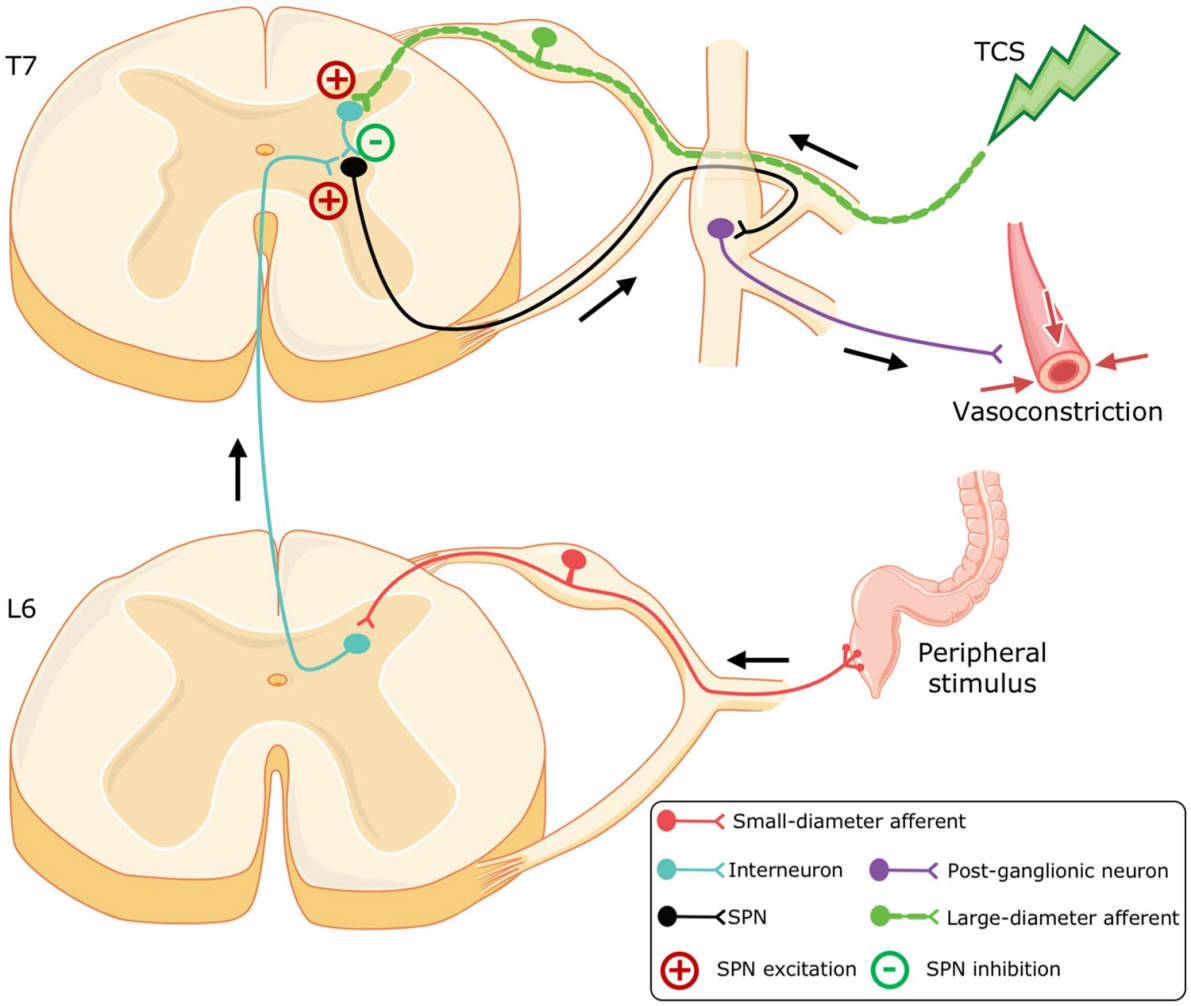
Known mechanisms of autonomic dysreflexia and theoretical framework of how TCS mitigates the dysfunction. Maladaptive small diameter sensory afferents (red) transmit noxious stimuli to ascending propriospinal interneurons (blue). The ascending signal activates thoracolumbar sympathetic preganglionic neurons (SPNs; black) and postganglionic neurons (purple) to trigger vasoconstriction in peripheral vasculature. This uncontrolled hypertensive response remains uninhibited due to the disruption of supraspinal control by SCI (not shown). Application of TCS results in activation of local large-diameter afferents and close the gates for noxious stimulus thereby preventing and/or interrupting the hypertensive response even in the presence of a noxious stimulus

Despite obvious methodological and biophysical differences, both TCS and epidural stimulation share remarkable mechanistic similarities. EMG evidence showing a high degree of congruency in evoked potentials suggests that both methods stimulate common neural correlates [37]. Mechanistically, spinal cord stimulation is derived from the ‘gate control theory’ [38]. At clinically used parameters, electrical stimulation predominantly recruits low-threshold, large-diameter proprioceptive, and cutaneous afferents (Fig. 8) [39]. Computer simulations suggest that during TCS, ~ 8% of the total current flows through the cerebrospinal fluid, generating high current densities along the posterior root afferents [39]. The non-noxious spinal input via large-diameter afferents can transsynaptically activate inhibitory interneurons, thereby ‘closing the gate’ for noxious stimuli (e.g., CRD and DARS) and preventing the uncontrolled reflex response (i.e., autonomic dysreflexia; Fig. 8). While this tends to explain the effects of real-time TCS, the long-lasting effects of TCS are likely a result of the adaptive plasticity of cardiovascular neural circuits—a hypothesis worthy of further comprehensive physiological and neuroanatomical research.

A limitation of this study is the lack of detailed information on the type of cardiac arrhythmia observed during autonomic dysreflexia episodes. Since cardiac arrhythmias were identified in retrospect, ECG recordings were not available in support of the BP data. However, a previous study has shown identical BP traces in rats with SCI in response to CRD and has confirmed arrhythmia as atrioventricular blocks using ECG [40]. Future studies will also examine the minimum stimulation thresholds needed to elicit these therapeutic responses. Another noteworthy observation is that the experimental autonomic dysreflexia in the human participant was conservatively performed in a controlled manner, keeping the individual’s safety in consideration. The efficacy of TCS to mitigate more severe episodes of autonomic dysreflexia is yet to be investigated.

Despite the unanswered questions, electrical neuromodulation is among the most promising treatments for SCI. In addition to highlighting the need for more mechanistic studies, the present study warrants longitudinal clinical investigations for the safety and efficacy of TCS in cardiovascular dysfunction, with possible permeation into the recovery of other crucial autonomic functions, e.g., gastrointestinal and genitourinary systems.

## Supplementary Information

Below is the link to the electronic supplementary material.

Supplementary file1 (PDF 517 KB)

Supplementary file2 (PDF 526 KB)

Supplementary file3 (PDF 534 KB)

Supplementary file4 (PDF 543 KB)

Supplementary file5 (PDF 543 KB)

Supplementary file6 (PDF 543 KB)

Supplementary file7 (PDF 543 KB)

Supplementary file8 (PDF 543 KB)

Supplementary file9 (PDF 526 KB)

## Data Availability

The pertinent data has been carefully documented within this manuscript. Methods described in brief have been referenced to the published studies for further detail. Raw data that is not provided in the article will be made directly available upon request by any qualified investigator.
